# Supplementary material for: Selective CDK9 Inhibition by Natural Compound Toyocamycin in Cancer Cells
Source: Cancers (Basel). 2022 Jul 8;14(14):3340. doi: 10.3390/cancers14143340 (PMC9324262; doi:10.3390/cancers14143340)
Supplement: Supplementary file 1 [file cancers-14-03340-s001.zip › cancers-1732088-supplementary.pdf]

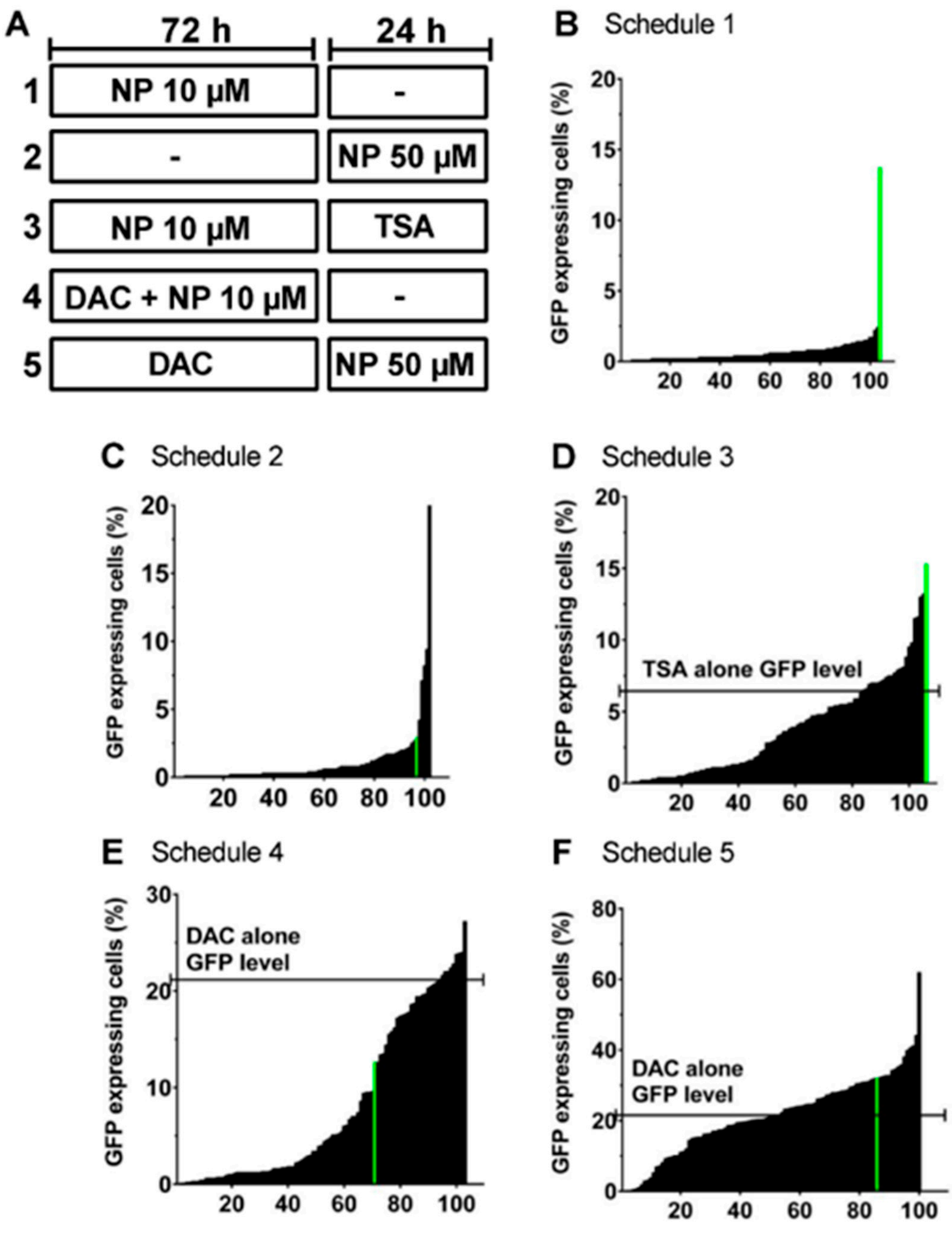

Figure S1. (**A**) Scheme depicting the 5 dose schedules used for the drug screen. (**B–F**) Graphs showing the percentage of GFP-expressing YB5 cells against all the 120 natural compounds tested in the screen. Horizontal lines show the GFP-expressing values obtained after TSA or DAC treatments alone. Green bars show the value obtained for toyocamycin.

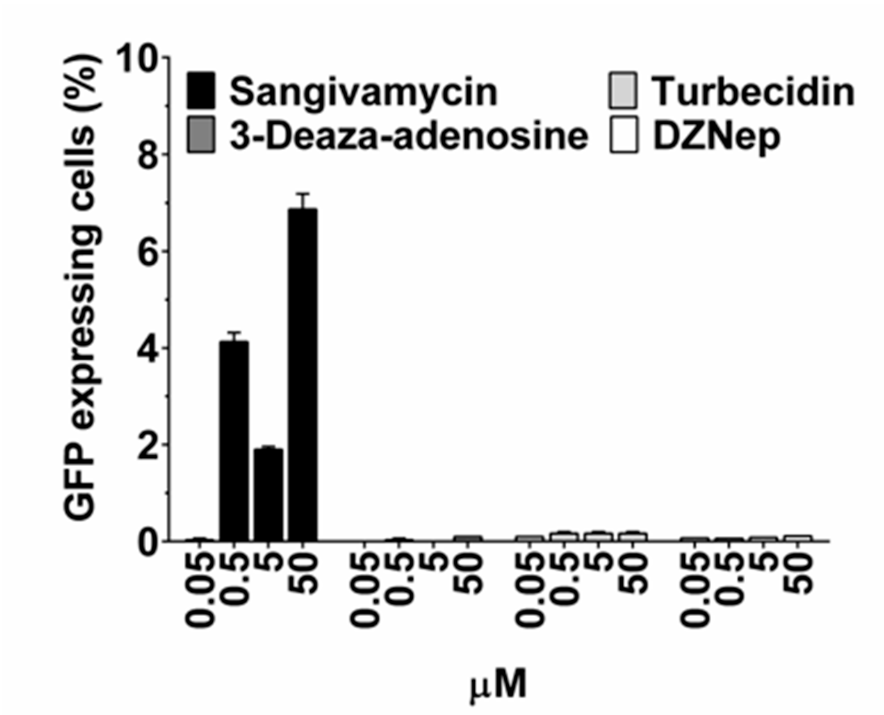

Figure S2. Graphs showing the percentage of GFP-expressing YB5 cells after the treatment with sangivamycin, tubercidin, 3-deaza-adenosine, and DZNep for 24 h. Doses are indicated on the graph.

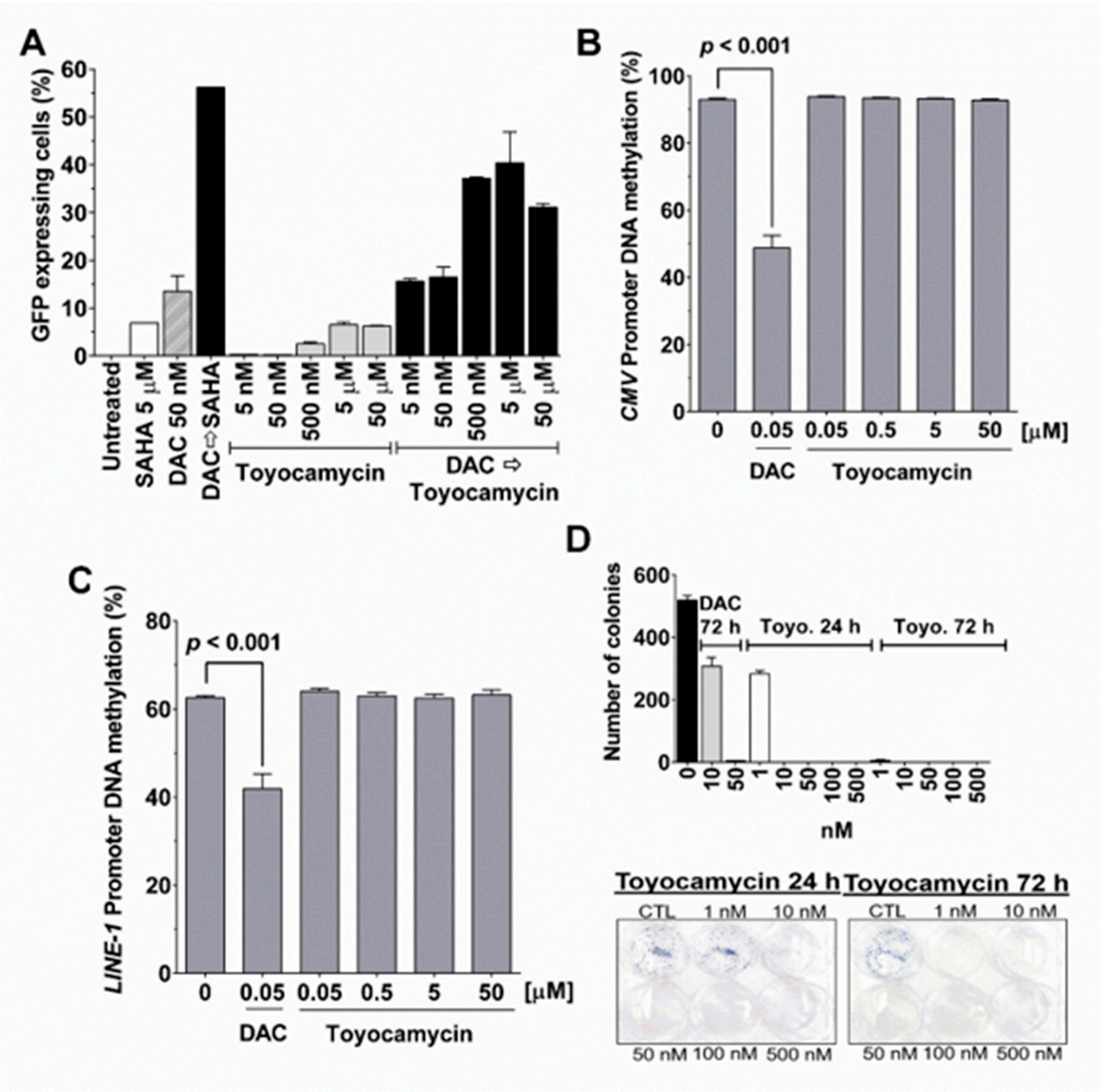

Figure S3. Toyocamycin synergizes with DAC treatment and is a potent anticancer agent. (**A**) Percentage of GFP expressing YB5 cells after treatments with SAHA, DAC, toyocamycin and their sequential combination (at doses indicated on the graph). (**B–C**) DNA methylation analysis of CMV and LINE-1 promoter regions by pyrosequencing following DAC and toyocamycin treatments (n = 3, p-values are indicated on the graph). (**D**) Loss of clonogenicity after DAC and toyocamycin treatments in YB5 cells (n = 3).

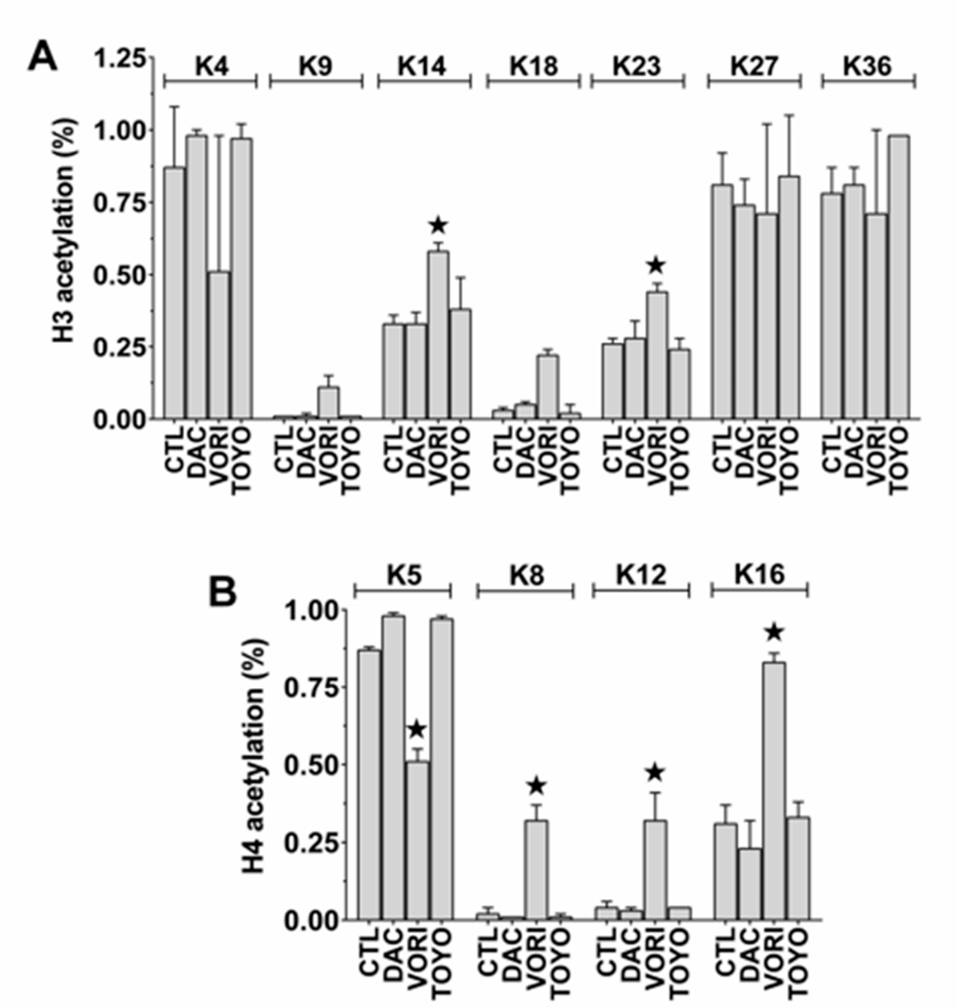

Figure S4. Histone acetylation levels in YB5 cells (untreated control, CTL) and after DAC treatment, SAHA (vorinostat, Vori), and toyocamycin (Toyo). Histone acetylation was measured on (**A**) histone 3 (H3) and on (**B**) histone 4 (H4).

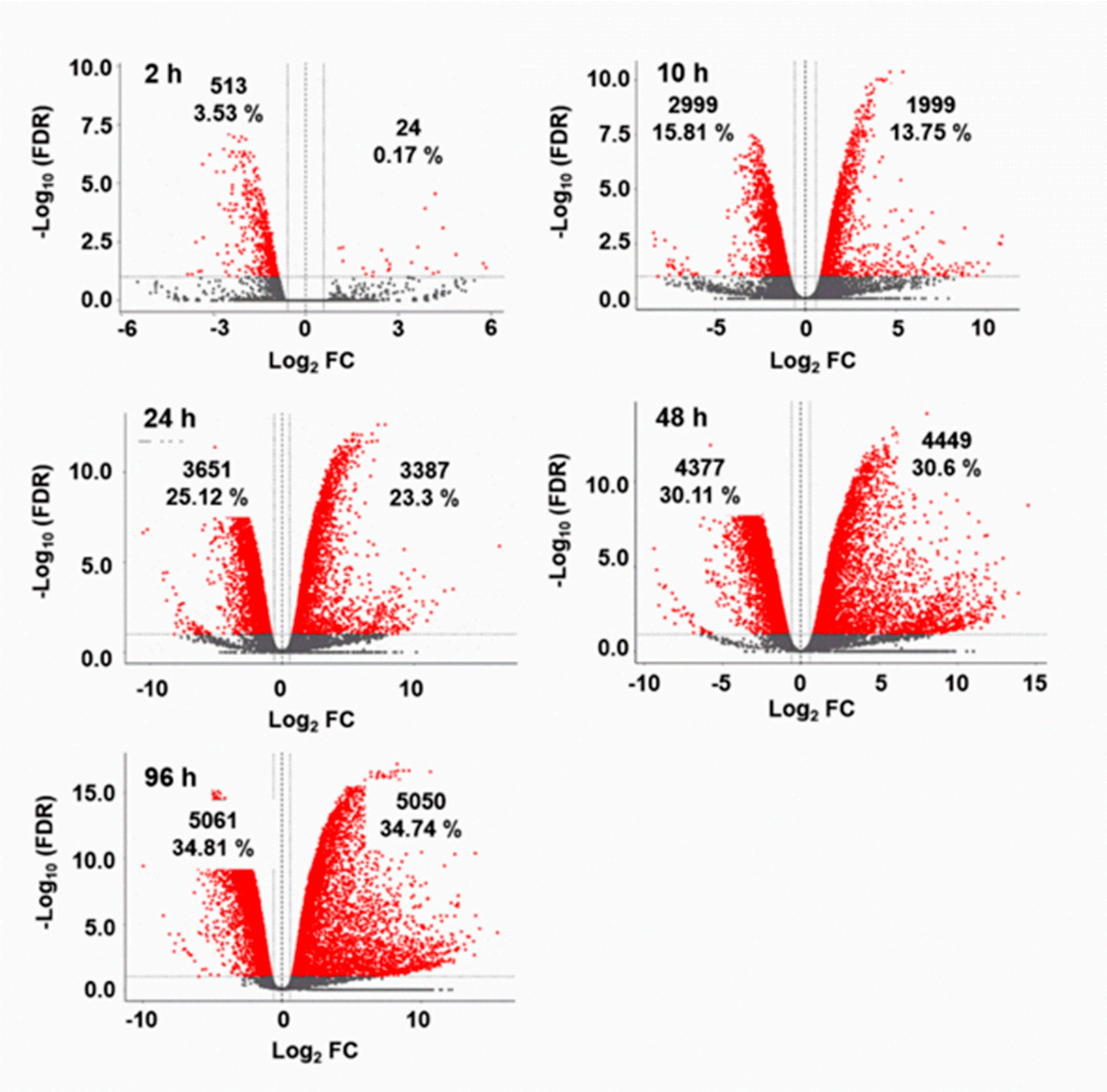

Figure S5. Volcano plots showing differential gene expression after toyocamycin treatment (250 nM) for 2 h, 10 h, 24 h, 48 h, and 96 h. Differentially expressed genes with a significant *p* adjusted values are shown in the graph with their respective percentages.

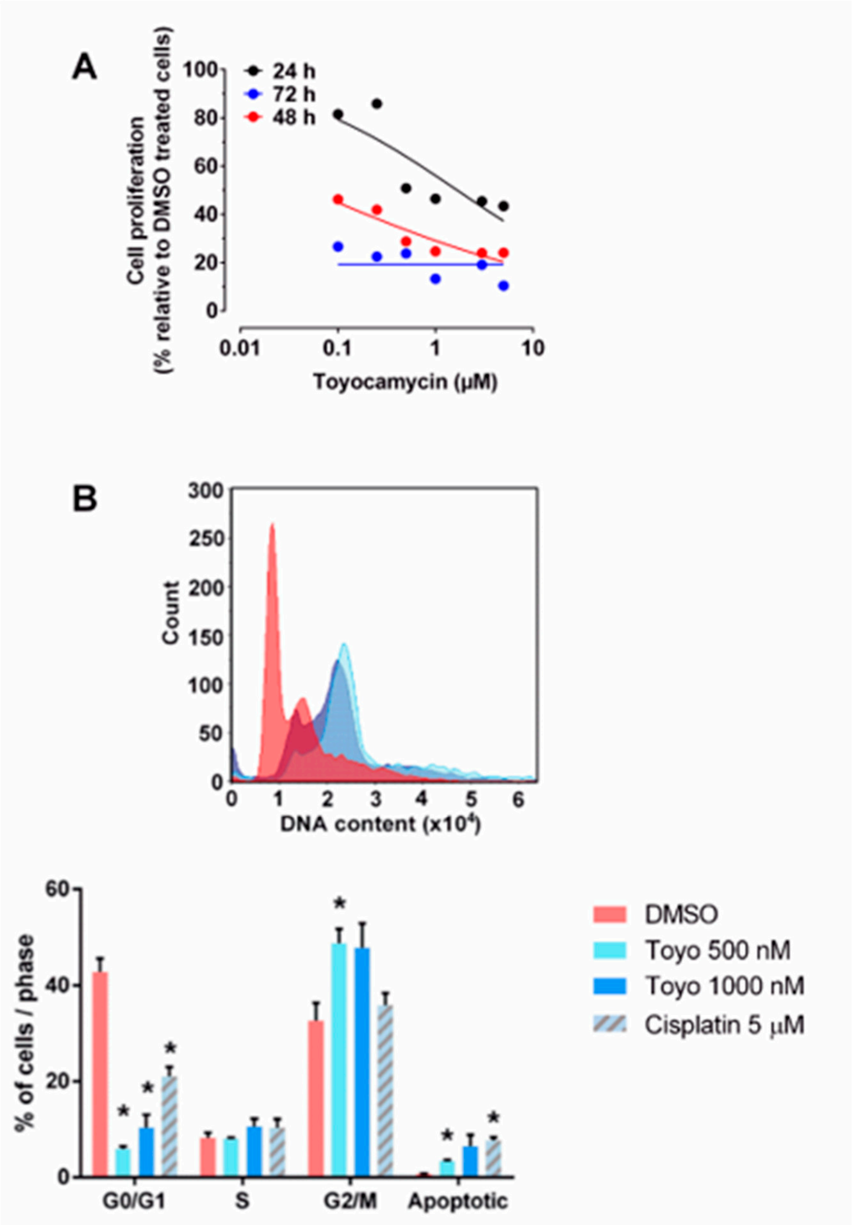

Figure S6. (**A**) Melanoma cell (451-Lu-BR cells) proliferation analysis after toyocamycin treatment (24, 48, and 72 h) at the doses indicated on the graph relative to untreated cells ($n = 3$). (**B**) Cell cycle analysis in HCT116 cells after toyocamycin treatment (500–1000 nM) for 24 h. Cisplatin treatment is used as a control for cell cycle block. Top graph shows the flow cytometry plots of DNA content and counts. Bottom graph shows the percentage of cells in each phase of the cell cycle ($n = 3$, a star indicates statistical difference between treated group and control, ANOVA, $p < 0.05$).

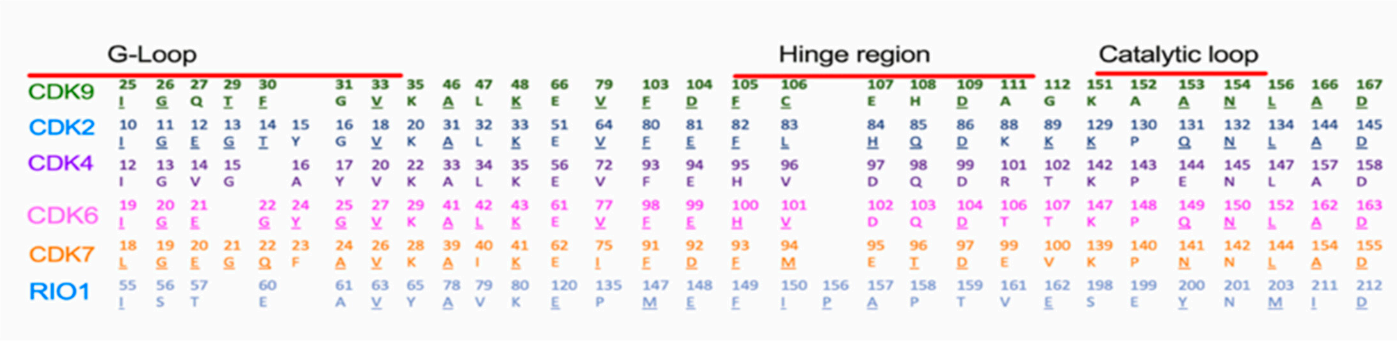

Figure S7. Sequence alignment of the ATP binding site residues obtained after superimposing the protein structures using the binding site residues and the match align command of UCSF Chimera. G-Loop, Hinge Region, and Catalytic Loop are shown in the graph.

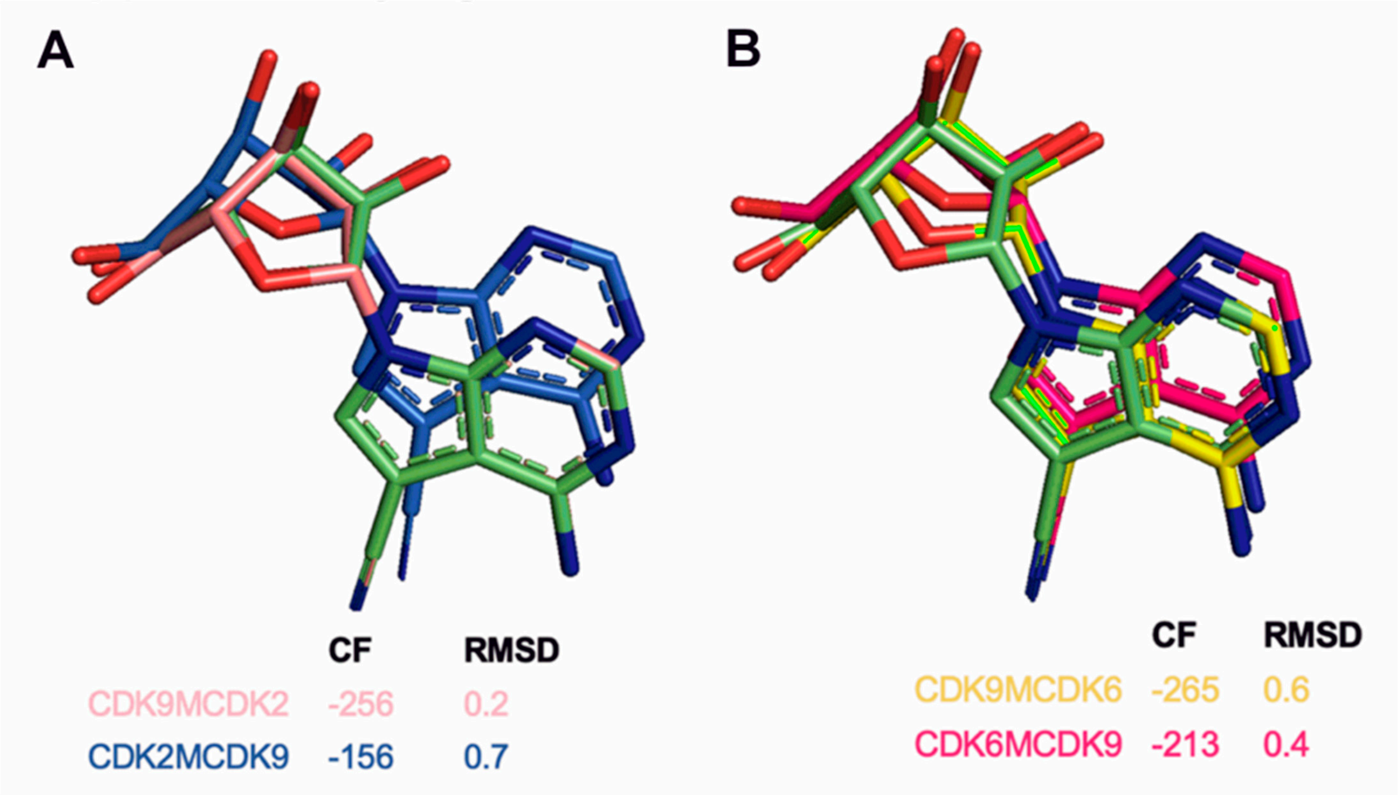

Figure S8. Mutational analyses between CDK9, CDK2, and CDK6 demonstrate the involvement of catalytic site and protein backbone for toyocamycin activity.

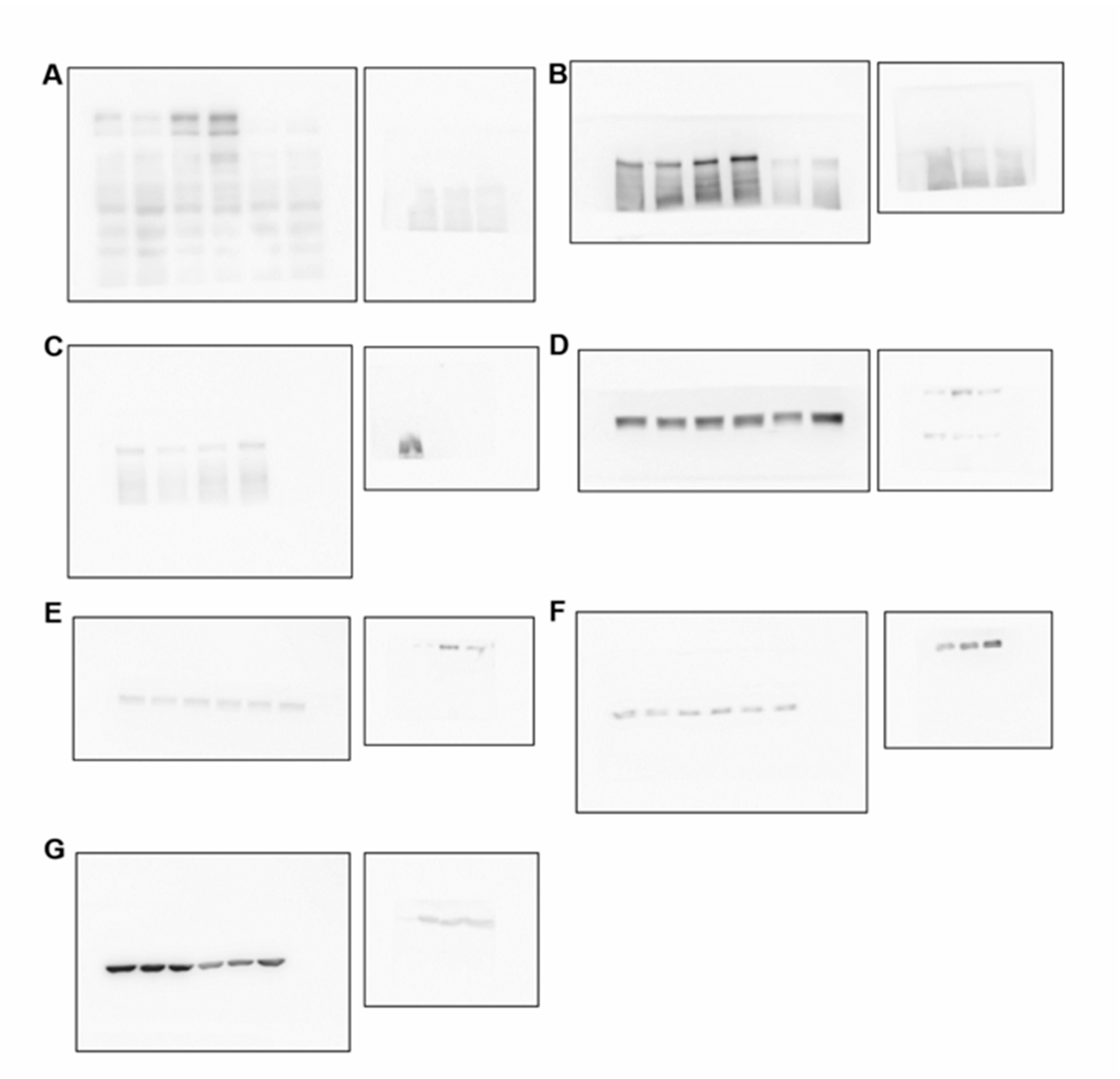

Figure S9. Full western blot images from Figure 5 (**A**) RNA-Pol II (N-term); (**B**) RNA-Pol II (C-term), (**C**) phospho-Pol II, (**D**) Rb, (**E**) phospho-Rb, (**F**) CDK9 and (**G**) β-actin. Left images correspond to toyocamycin treatment, right images correspond BAY 1251152 treatment.

Table S1. Connectivity mapping performed on RNA-seq results obtained in YB5 cells after 10 h exposure to 250 nM toyocamycin**.**

Rank	Cmap drug name	Primary Target	*p*-value	References
1	Alsterpaullone	CDK1	0.00000	[34]
2	Cephaeline	Protein synthesis inhibitor	0.00000	[35]
3	Camptothecin	DNA topoisomerase 1	0.00000	[39]
4	Emetine	Protein synthesis inhibitor	0.00000	[35]
5	GW-8510	CDK2-5	0.00000	[36,37]
6	Azacitidine	DNA methyltransferase	0.00000	[40]
7	Irinotecan	DNA topoisomerase 1	0.00000	[41]
8	Thioguanosine	DNA, RNA synthesis	0.00000	[42]
9	H-7	PKC / CDK2	0.00000	[37,38)
10	0175029-0000	CDK	0.00000	[37]

Table S2. Root Mean Square Deviation (RMSD) values between the different CDKs and Rio1 crystal structure after superimposition of the binding site residues, RMSD values are in Angström (Å).

	**CDK2**	**CDK6**	**RIO1**	**CDK4**	**CDK7**	**CDK9**
**RIO1**	4.8	4.7	0	4.5	4.4	4.4
**CDK9**	1.3	1.4	4.4	1.5	1.3	0

Table S3. Summary of the docking simulations, CF values are in AU (arbitrary unit). Docking simulation results showing different parameters, REF_CF (CF value of Rio1 crystal pose), RMSD (Best RMSD from the reference position), CF (corresponding CF value of the best RMSD pose), Best_CF and Best_CF_RMSD.

	RIO1	CDK9	CDK7	CDK6	CDK2	CDK6MCDK9	CDK9MCDK6	CDK9MCDK2	CDK2MCDK9	CDK4
**REF_CF**	−192.2	−125.3	+	+	+	+	−257.3	−250.3	+	−72
**CF**	−199.9	−148	−151.9	−40.9	−156	−213	−265	−256	−156	−64
**RMSD**	0.42	1.5	1.14	1.66	2.29	0.4	0.6	0.2	0.7	1
**Best_CF**	−212.7	−195.2	−162.5	−195.4	−186.2	−228	−294	−259.9	−247.6	−152.9
**Best_CF_RMSD**	1	3.9	3.2	4.5	3	5.2	0.9	0.7	2	3.5
